# Electrocardiographic Characteristics and Catheter Ablation of Ventricular Arrhythmias Originating From the Moderator Band in Children

**DOI:** 10.3389/fped.2022.740230

**Published:** 2022-02-09

**Authors:** Diandong Jiang, Jianli Lv, Bo Han, Xiaofei Yang, Lijian Zhao, Yingchun Yi, Deyong Long, Caihua Sang

**Affiliations:** ^1^Department of Pediatric Cardiology, Shandong Provincial Hospital Affiliated to Shandong First Medical University, Jinan, China; ^2^Department of Cardiology, Beijing Anzhen Hospital, Capital Medical University, Beijing, China

**Keywords:** ventricular arrhythmias, moderator band, catheter ablation, electrocardiographic morphology, electrophysiological characteristics

## Abstract

**Aims:**

To investigate the electrocardiographic (ECG) characteristics and catheter ablation of ventricular arrhythmias (VAs) originating from the moderator band (MB) in children.

**Methods:**

A total of six children who had VAs originating from the MB—as confirmed by electrophysiological study—and who underwent catheter ablation between January 2016 and December 2020 were retrospectively reviewed. During the procedure, a three-dimensional electroanatomic mapping system was used to facilitate three-dimensional anatomical reconstruction, mapping and ablation. Patients' clinical characteristics, ECG features and procedural data were collected and analyzed.

**Results:**

The mean age was 8.4 ± 2.6 years (range: 5.3–11 years) and mean weight was 27.7 ± 11.4 kg (range: 17–47 kg). Four patients presented with frequent premature ventricular contraction (PVC), one patient presented with frequent PVC and non-sustained ventricular tachycardia, and one patient presented with sustained monomorphic ventricular tachycardia. The QRS duration averaged 126.3 ± 4.6 ms. In all patients, the VAs had left bundle branch block QRS with left superior frontal plane axes, rapid downstrokes of the QRS in the precordial leads, and late precordial transitions (>V_4_). During the same period, 10 cases of VAs originated from the posterior-lateral wall of the tricuspid annulus, with a mean QRS duration of 152.8 ± 6.4 ms. Compared to that, VAs of MB origin have narrower QRS widths, downstroke slopes in the inferior lead, sharper downstroke slopes in the precordial lead, and smaller R-wave amplitudes in the V_6_ lead. All patients experienced immediate ablation success with activations earlier than QRS by 26.0 ± 3.5 ms, and no procedural complications occurring. Only one case had recurrent PVC during a follow-up period ranging from 6 to 36 months.

**Conclusion:**

MB VAs in children have distinctive ECG morphology and electrophysiological characteristics. Catheter ablation using a three-dimensional electroanatomic mapping system is safe and effective in these patients.

## Introduction

The moderator band (MB) of the right ventricle (RV) is a muscular structure containing Purkinje fibers that extend from the septum to the free wall of the RV (1). Ventricular arrhythmias (VAs) originating from the MB are rare in children, and relevant clinical experience and research on them are limited. VAs that originate in the MB are characterized by their specialized anatomical structures and distinct electrophysiological and electrocardiographic features. Additionally, the mapping and catheter ablation of MB VAs remains challenging. In this study, we described the electrocardiographic characteristics, mapping and ablation in children with VAs originating from the MB.

## Materials and Methods

### Patient Population

A total of six children with premature ventricular contraction (PVC) or ventricular tachycardia (VT) that originated from the MB and underwent ablation between January 2016 and December 2020 were enrolled in the study. All patients underwent routine pre-procedural evaluations, including notation of myocardial injury markers, electrocardiograms (ECGs), chest X-rays, 24 h Holter monitoring and transthoracic echocardiography (TTE). Structural heart disease was excluded as an underlying factor in all patients. The study was approved by the Ethics Committee of Shandong Provincial Hospital Affiliated to Shandong First Medical University. All procedures complied with the Declaration of Helsinki, and parents provided informed consent.

### Electrocardiographic Analysis

Twelve-lead surface sinus rhythm ECGs, and VAs, were recorded before the procedure. The morphology, duration, amplitude and precordial vector transition of VA QRS were analyzed.

### Electrophysiologic Study and Radiofrequency Ablation

**Figure 1:**
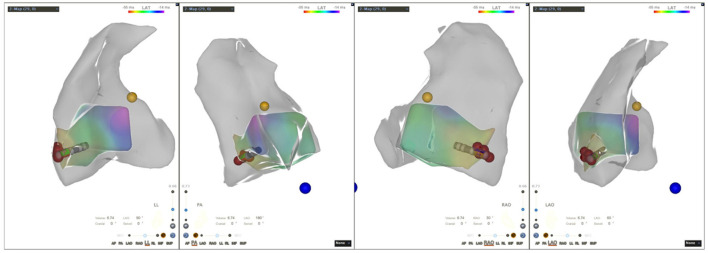
Three-dimensional electroanatomic model of the right ventricle (RV-LL-PA-RAO-LAO). RV, right ventricle; LL, left lateral; PA, posteroanterior; RAO, right anterior oblique; LAO, left anterior oblique.

**Figure 2:**
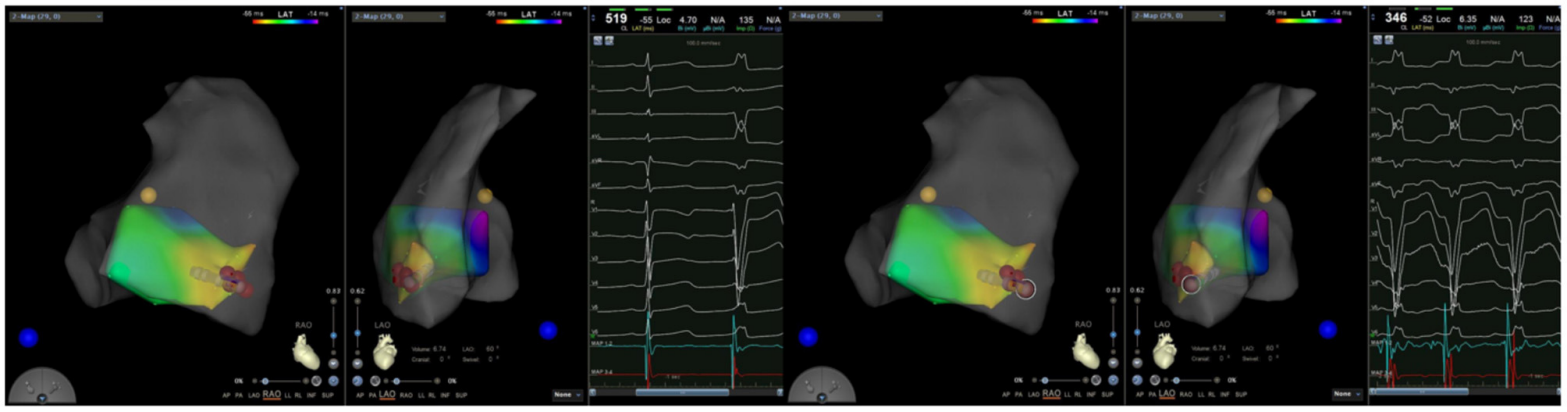
(Left) Activation mapping; (Right) Pace mapping is used to verify that the morphology of premature ventricular contraction is consistent with surface electrocardiogram.

All patients discontinued antiarrhythmic drugs at least five half-lives before the procedures. VAs were preliminarily determined to have originated from the RV using 12-lead ECG. The procedure was performed under general anesthesia in all six cases. An 8.5 F Swartz sheath was placed in the right atrium to assist in catheter manipulation and increase its stability. A CARTO electroanatomic mapping system (Biosense Webster, Diamond Bar, CA, USA) was used to guide the electroanatomic mapping, and three-dimensional anatomic reconstruction of the RV was performed (Figure 1). A 3.5-mm irrigated-tip catheter (ThermoCool SmartTouch, Biosense Webster, Diamond Bar, CA) was used for mapping and ablation. Activation mapping was performed during the onset of VA (Figure 2) and further detailed activation mapping was performed to determine the earliest activity. The site of origin was also verified using pace mapping (Figure 2), and the ablation target was selected in the site where the QRS activation morphologies and pace mapping were consistent with those of clinical VAs. If no VAs were noted, isoproterenol infusion and/or burst pacing from the RV were performed to provoke VAs.

Radiofrequency energy was applied at a power of 30–35 W and a maximal temperature of 45°C. If PVC/VT termination occurred within the first 10 s, the site was regarded to be an effective ablation target, and radiofrequency delivery continued for 60–120 s, followed by local ablation near the target. If not, the ablation was terminated and the target was retargeted. Immediate ablation success was defined as the absence of spontaneous or inducible PVC/VT with repeated isoproterenol infusion and/or RV pacing for at least 30 min after final elimination of the VA. If PVC/VT was observed again during a minimum 30-min observation period, re-ablation at the original target with increased radiofrequency energy or re-mapping to a new target was performed.

### Follow-Up

After the procedure, continuous ECG telemetry monitoring was performed for 24 h. All patients had ECG, TTE and 24 h Holter monitoring for 1 day after the procedure, and were then scheduled for follow-ups at 1, 3, 6, 12, 24, and 36 months after the procedure. During the follow-up, the absence of VT/PVC, or a reduction of more than 75% in the total number of PVCs with the same morphology compared to pre-procedural results on 24 h Holter monitoring, was considered an indication of long-term success.

### Statistical Analysis

Statistical analysis was performed using SPSS software version 23.0 (SPSS Inc., Chicago, IL). Categorical data were expressed as counts and/or percentages, and continuous variables were presented as means ± standard deviation (SD). Comparisons were performed using χ^2^ tests and Student's *t*-tests. *P*-values < 0.05 were considered statistically significant.

## Results

### General Characteristics

From January 2016 to December 2020, 161 consecutive children with VAs at our center underwent radiofrequency ablation with a three-dimensional electroanatomic mapping system. Ninety-six cases originated from the RV. The immediate success rate was 96.9% (156/161), and the recurrence rate was 6.8% (11/161). Six patients from this cohort presented with VAs originating from the MB. Of these cases, three were males. The mean age was 8.4 ± 2.6 years (range: 5.3–11 years) and mean weight was 27.7 ± 11.4 kg (range: 17–47 kg). Four patients presented with frequent PVC, one patient presented with frequent PVC and non-sustained VT, and one patient presented with sustained monomorphic VT. Pre-procedural hypersensitive troponin T and N-terminal brain natriuretic peptide precursors were normal in all patients, as were cardiac sizes and left ventricular ejection fraction on TTE. Further, no evidence of organic heart disease was found. All patients had taken at least one antiarrhythmic medication, and were undergoing radiofrequency ablation for the first time. Patient characteristics are shown in Table 1.

**Table 1:** General characteristics.

**Case**	**Age (years)**	**Sex**	**Weight (kg)**	**LVEF (%)**	**Clinical arrhythmia**	**PVC burden (%)**	**Antiarrhythmic medication**
1	11	Male	33	64	PVC/NSVT	14.1	Propafenone
2	11	Male	47	63	PVC	21.1	Propafenone
3	5.3	Female	17	65	PVC	25.2	Propafenone, Sotalol
4	5.8	Female	19	64	PVC	11.0	Propafenone
5	10	Female	30	64	PVC	12.1	Propafenone
6	7	Male	20	64	SMVT	–	Propafenone, Amiodarone

*LVEF, left ventricular ejection fraction; PVC, premature ventricular contraction; NSVT, non-sustained ventricular tachycardia; SMVT, sustained monomorphic ventricular tachycardia*.

### Electrocardiographic Characteristics

**Figure 3:**
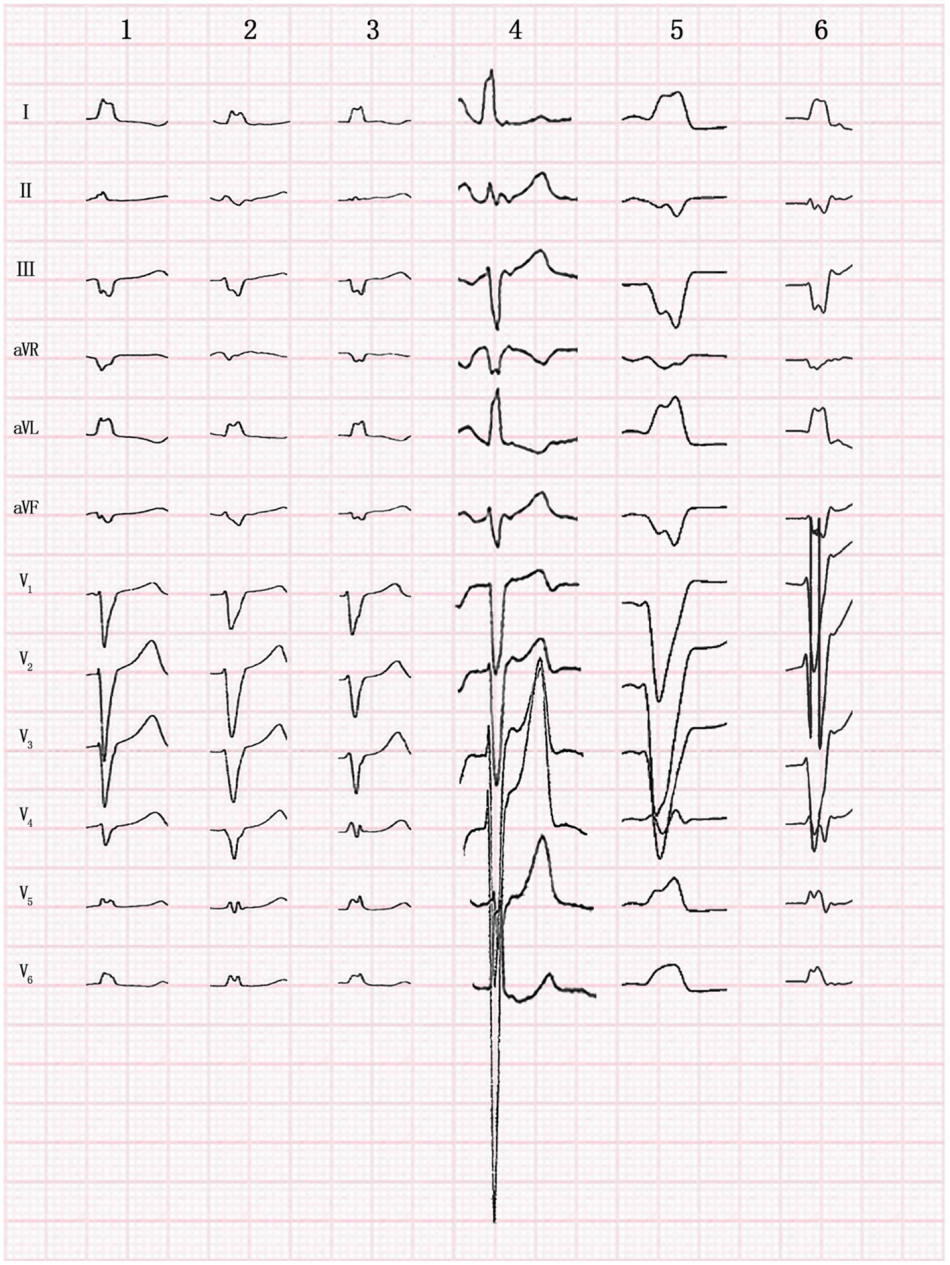
Twelve-lead morphology of premature ventricular contraction originating from the moderator band in 6 patients.

**Figure 4:**
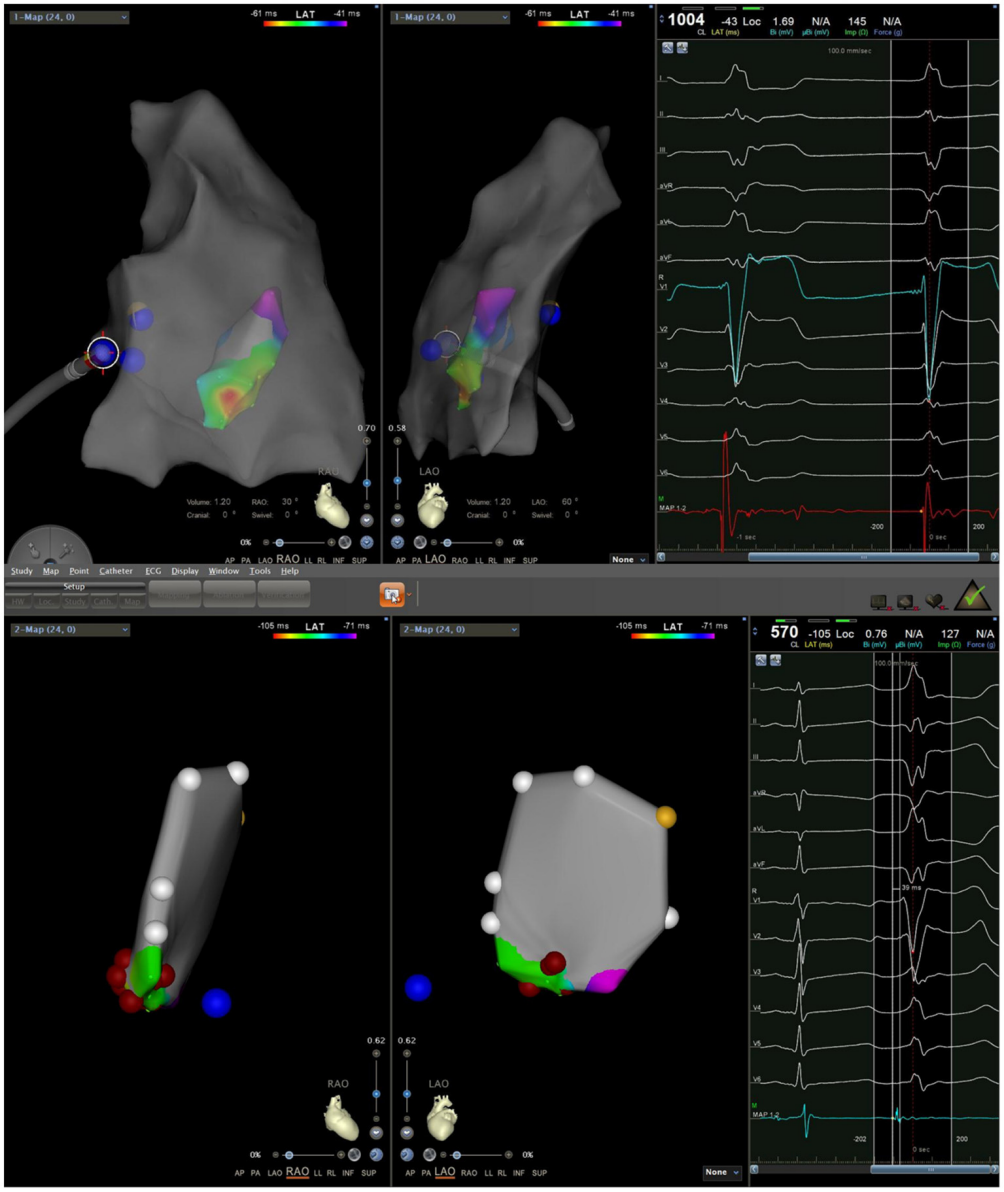
Electrocardiogram (ECG) comparison between premature ventricular contraction (PVC) originating from the moderator band (MB) and the posterior-lateral wall of tricuspid annulus. In the figure above, the left ECG was MB origin, while the right was origin of the posterior lateral wall of the tricuspid annulus by pacing. In the figure below, the ECG was spontaneous PVC originating from the posterior-lateral wall of tricuspid annulus. PVC of MB origin has a sharper slope of downstroke in the precordial lead.

All patients presented with normal sinus rhythms, and the QRS morphologies of clinical VAs were recorded using an ECG before all procedures (Figure 3). Electrocardiographic characteristics are listed in Table 2. The mean QRS duration during VA was 126.3 ± 4.6 ms (range: 120–132 ms), suggesting a pattern of left bundle branch blocks. The VAs had fast downstroke slopes at the QRS in the precordial lead, with precordial transition later than lead V_4_. Precordial transitions were later for the VAs than for sinus rhythms, and the QRS amplitudes of lead V_6_ were lower than those of sinus rhythms. VA morphologies included left superior axes, with positive QRS complexes in leads I and aVL, and negative QRS complexes in leads III and aVF. Downstroke slopes at the inferior lead QRS had notches, and the aVR leads QRS complexes were negative, with low amplitude and stumbles. During the same period, 10 cases of VAs originated from the posterior-lateral wall of the tricuspid annulus, with a mean QRS duration of 152.8 ± 6.4 ms (range: 142–162 ms). Compared with the VAs originating from the posterior-lateral wall of the tricuspid annulus, QRS duration in VAs that originated from the MB was significantly narrower (*P* = 0.001) (Figure 4).

**Table 2:** Electrocardiographic characteristics.

**Case**	**Baseline ECG**	**Axis**	**Morphology**	**QRS duration during arrhythmia (ms)**	**Transition during arrhythmia**	**Transition in sinus rhythm**
1	Normal	Left superior	LBBB	126	V_5_	V_3_
2	Normal	Left superior	LBBB	130	V_5_	V_2_
3	Normal	Left superior	LBBB	122	V_5_	V_3_
4	Normal	Left superior	LBBB	120	V_6_	V_2_
5	Normal	Left superior	LBBB	128	V_5_	V_4_
6	IRBBB	Left superior	LBBB	132	V_5_	V_3_

*ECG, electrocardiogram; LBBB, left bundle branch block; IRBBB, incomplete right bundle branch block*.

### Mapping and Ablation

**Figure 5:**
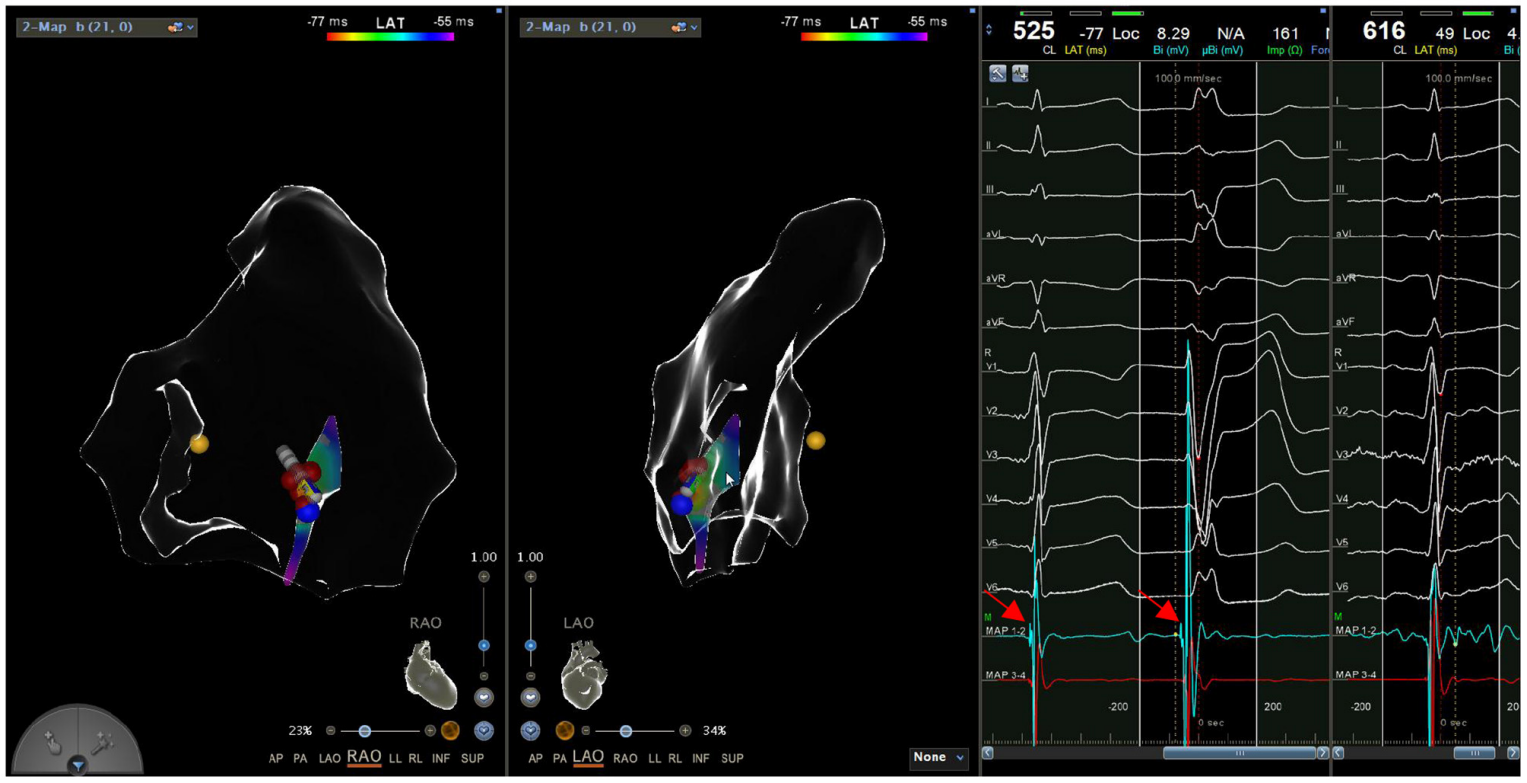
A Purkinje potential (arrow) is recorded in both sinus rhythm and PVC in a continuous way.

During the procedure, all six patients had spontaneous PVC/VT. Activation mapping was used to confirm ablation targets. The mean local activation at the sites of successful ablation was 26.0 ± 3.5 ms (range: 22–32 ms) before the onset of the PVC/VT at the QRS complex on the surface ECG. A Purkinje potential at the effective ablation site which preceded the ventricular activation during PVC was found in three patients. This potential was also noted in sinus rhythm (Figure 5). The mean ablation time was 165 ± 52.8 s (range: 90–240 s) and mean procedure time was 81.7 ± 31.3 min (range: 60–140 min). Among the 6 cases, 4 cases did not receive radiation at all, and the fluoroscopic time of the other 2 cases was no more than 1 min, with radiation dose of 1 mGy, respectively. Immediate ablation success without any complications was achieved in all cases (Table 3).

**Table 3:** Ablation and follow-up.

**Case**	**Purkinje potential**	**Pre-PVC (ms)**	**Ablation time (s)**	**Procedure time (min)**	**Fluoroscopic time (min)**	**Radiation dose (mGy)**	**Immediate success**	**ECG post-procedure**	**Follow-up duration (months)**	**Recurrence**
1	Yes	25	180	60	0.2	1	Yes	Normal	6	No
2	No	25	90	60	0	0	Yes	Normal	12	No
3	No	22	120	60	0	0	Yes	Normal	12	Yes
4	Yes	24	240	90	0	0	Yes	CRBBB	24	No
5	Yes	28	180	80	0	0	Yes	Normal	24	No
6	No	32	180	140	1	1	Yes	CRBBB	36	No

*PVC, premature ventricular contraction; ECG, electrocardiogram; CRBBB, complete right bundle branch block*.

### Follow-Up

The patients were followed for 6–36 months after their procedures. A 5-year-old girl with PVC burden of 25.2% had recurrent PVC 1 month after the procedure. The procedure was acutely successful. The patient responded poorly to two antiarrhythmic drugs (propafenone and sotalol) before the procedure, but responded well to a single antiarrhythmic drug (propafenone) after the procedure. The case didn't undergo a second procedure for arrhythmia recurrence. There was no recurrence of PVC/VT in the other five patients.

## Discussion

VAs are common arrhythmias in children. Of these arrhythmias, PVC/VT that originate from the RV are the most frequent (2). The majority of VAs originating from the RV originate from the right ventricular outflow tract and tricuspid annulus (3, 4). MB origin, however, is rare– accounting for just 6.3% of the RV origins in the present study. As such, there are few reports on VAs that originate from MB. To our knowledge, this is the first study on MB-originating VAs in children.

The MB is part of the septomarginal trabeculation, which extend across the RV. It is a muscular structure that connects the septum to the free wall of the RV. The MB emanates from the septomarginal trabeculation on the septal side. It attaches to the septal papillary muscle and to the anterior superior papillary muscle on the free wall side. The MB contains the RV conduction system (a Purkinje fiber bundle) which cross the endocardium from the interventricular septum and spreads to the RV free wall, supplying the subendocardial ventricular plexus (1). The physiological function of the MB is mainly to direct blood to the pulmonary artery during systole and to prevent RV hyperdilation during diastole. The morphology of the MB varies, showing significant diversity in size, length, and insertion origin. Origin and insertion origins differences may be the main drivers of morphological variety on ECGs (5). The MB is also supplied by nerve fascicles containing cholinesterase. These characteristic morphological and structural features of the MB may be associated with the occurrence of arrhythmias.

Our findings are similar to those noted in a former study on 10 adults with idiopathic VAs originating from the MB (6). All patients with MB arrhythmias had left bundle branch block morphologies with left superior axes. MB VAs have a relatively narrow QRS duration, and a large downstroke QRS slope in the precordial lead, with transitions that are later than both lead V_4_ and sinus QRS. VAs morphologies generally have positive QRS complexes in the inferior lead, with downstroke QRS slopes. The QRS complex of the I and aVL leads is positive, and the aVR lead is negative, with low amplitude and stumbles. The electrocardiographic characteristics of MB VAs were similar to those of VAs originating from the posterior-lateral wall of the tricuspid annulus. Thus, MB VAs are easily misdiagnosed as originating from the tricuspid annulus. However, careful identification reveals some distinction in the ECG signatures of the two origins. VAs of MB origin have narrower QRS widths, downstroke slopes in the inferior lead, sharper downstroke slopes in the precordial lead, and smaller R-wave amplitudes in the V_6_ lead. VAs originating from the posterior-lateral wall of the tricuspid annulus, however, have negative QRS complexes in lead II and later precordial transitions. These electrocardiographic differences may be closely related to the unique anatomical location and electrophysiological features of the MB. Electrical activation of MB VAs can simultaneously spread to the septal free walls of the RV, with less time required for RV activation and better synchronization of RV depolarization compared to VAs of tricuspid annular origin.

It has been found that it is easy to induce ventricular fibrillation in PVCs of MB origin in patients with normal heart structures (7). The role of the Purkinje conduction system in triggering idiopathic ventricular fibrillation has also been previously demonstrated. Possible explanations for this phenomenon include that Purkinje cells are more prone to occur earlier post-depolarization than other regions of the ventricular myocardium, and that Purkinje fibers may lose intrinsic protective mechanisms due to tissue refractoriness abnormalities (8, 9). Thus, it may be beneficial for patients with idiopathic ventricular fibrillation to record PVCs that may have MB origins, in order to use it as a potential ablation target. In some patients with idiopathic ventricular fibrillation, empiric MB ablation can eliminate arrhythmias (6). Among the six patients in our study, one had inducible sustained VT, and one had inducible non-sustained VT. No PVC-induced ventricular fibrillation occurred.

In our study, ablations were based on the earliest PVC activities as determined by activation mapping, and ablation targets were further confirmed by pace mapping in sinus rhythm. In general, ablation of VAs can be performed based on pace-mapping in sinus rhythm to identify the origin if there is no inducible PVC/VT during the procedure. However, due to the similar QRS morphology of each segment of the MB, simple pace-mapping has limited value in determining the origin of MB VAs. Therefore, the mapping and ablation of this site should be performed using activation mapping when possible, and the accuracy of the ablation target can be further verified using a combination of activation mapping and pace mapping. A Purkinje potential can often be recorded at the earliest activation site during PVC or during sinus rhythm, and right bundle branch blocks are common after ablation. This is consistent with distribution of the right bundle branch, and also aligns with a study on the mechanisms of VT caused by the right bundle branch and Purkinje fibers at the MB site (6). Sadek et al. reported that PVC termination could be achieved by ablating the right bundle branch in one patient, but it was not observed in the remaining nine patients (6). We occasionally noted changes in QRS morphology during ablation, probably indicating a change in the exit site and eventual PVC elimination after extensive ablation. The site of successful ablation location along the MB was variable, including the septal insertion, the body of the MB, and the free wall insertion (6). Unfortunately, since we did not use intracardiac echocardiography (ICE), we could not further identify the exact site of successful ablation location along the MB.

As a part of the RV papillary muscle system, the MB is a beating structure in the cardiac cavity. The ablation success rate of MB VAs using three-dimensional electroanatomic mapping system alone is not high, but can be improved with ICE (10). However, ICE requires a minimum 11 F of vascular sheath, and so can rarely be used in children. In this study, a three-dimensional anatomic reconstruction of RV was performed, and a satisfactory target was mapped using the CARTO electroanatomic mapping system. All ablations were immediately successful, with Swartz sheaths supporting the ablation catheter. One of six patients had PVC recurrence, but this was still a significant improvement compared with pre-procedure rates. Low success and high recurrence are likely related to issues related to catheter-target contact or catheter stability. Despite adequate visualization with ICE, part of patients required a second procedure despite an acutely successful initial procedure (6). The potential mechanism for recurrence is challenging catheter contact and stability resulting in lower power delivery to the thick intracavitary structure.

None of these cases underwent cardic magnetic resonance imaging (MRI) prior and post the procedure. In our opinion, prior imaging (for example cardiac MRI) and RV segmentation or fibrosis segmentation may shorten procedure time, but seem to have little effect on reducing radiation exposure time due to the use of a three-dimensional electroanatomic mapping system. A post-procedure MRI may be helpful in determining the exact anatomic location of ablation. In the future, we plan to perform cardiac MRI prior and post the procedure for some patients with VAs of special site origin, especially to spot the anatomic location of ablation sites through post-procedure MRI.

VAs of MB origin are uncommon, and related reports are also rare. Although the sample size of this study was small, we described the ablation of VAs originating from the MB in children for the first time. The electrocardiographic and electrophysiological characteristics were consistent, and certain features were summarized for reference. Our results should be confirmed in a larger sample size. In this study, target localization mainly relied on the three-dimensional electroanatomic mapping system: no ICE was used to accurately map the ablation site on the MB. Thus, there were some limitations related to the anatomical localization of the target.

## Conclusion

VAs originating from the MB are rare, particularly in children, and have unique ECG and electrophysiological characteristics. Catheter ablation of such VAs is challenging. However, the clinical effects of mapping and ablation using a three-dimensional electroanatomic mapping system are satisfactory.

## Data Availability Statement

The raw data supporting the conclusions of this article will be made available by the authors, without undue reservation.

## Ethics Statement

Written informed consent was obtained from the individual(s), and minor(s)' legal guardian/next of kin, for the publication of any potentially identifiable images or data included in this article.

## Author Contributions

DJ and BH designed the study and performed the research. JL, XY, LZ, and YY performed the research and analyzed the data. DJ wrote the manuscript. DL and CS supervised the study. All authors contributed to manuscript revision, read, and approved the submitted version.

## Conflict of Interest

The authors declare that the research was conducted in the absence of any commercial or financial relationships that could be construed as a potential conflict of interest.

## Publisher's Note

All claims expressed in this article are solely those of the authors and do not necessarily represent those of their affiliated organizations, or those of the publisher, the editors and the reviewers. Any product that may be evaluated in this article, or claim that may be made by its manufacturer, is not guaranteed or endorsed by the publisher.
